# Supplementary material for: Genome-Wide Identification and Expression Analysis of WRKY Transcription Factors in Akebia trifoliata: A Bioinformatics Study
Source: Genes (Basel). 2022 Aug 26;13(9):1540. doi: 10.3390/genes13091540 (PMC9498614; doi:10.3390/genes13091540)
Supplement: Supplementary file 1 [file genes-13-01540-s001.zip › Supplementary figures.pdf]

Supplementary Figures

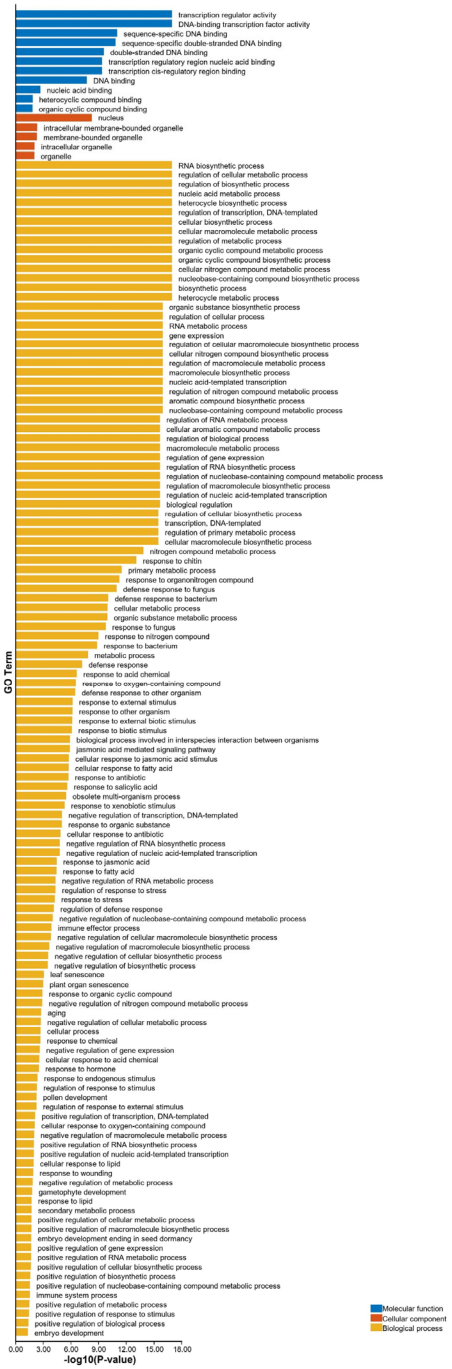

Figure S1. GO enrichment analysis map of the *WRKY* gene family. Blue blocks represent molecular functions, orange blocks represent cellular components, and yellow blocks represent biological processes.

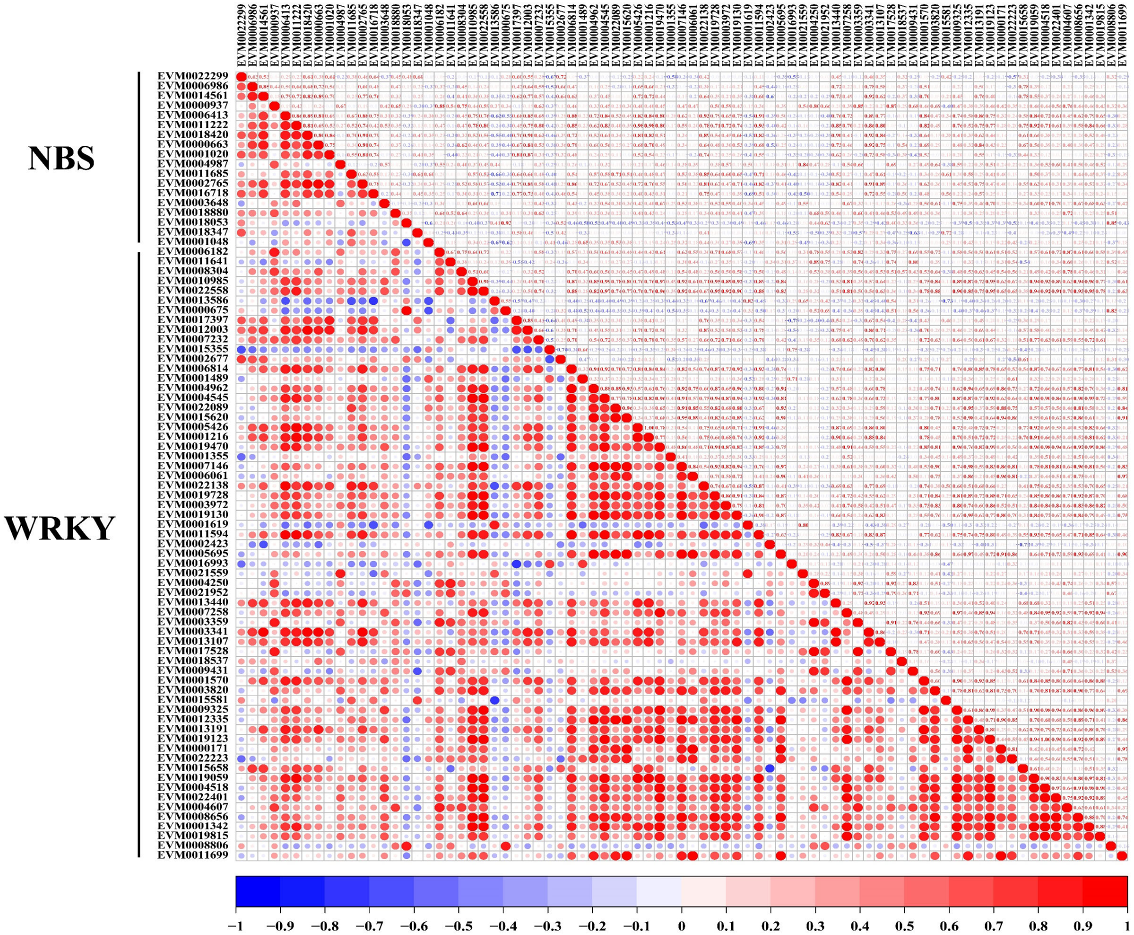

Figure S2. Correlation between 63 *AkWRKY* genes and 18 *AkNBS* genes.
